# Point-of-Care Ultrasound Detects Rapid Muscle Loss in Pediatric ECMO Patients—A Secondary Analysis Paper

**DOI:** 10.3390/pediatric18030063

**Published:** 2026-05-01

**Authors:** Mohammad Sabobeh, Elizabeth Seewer, William Hunt Stafford, Nicolas Chiriboga, Thomas Spentzas, Shyam Popat, Alyssa Clark, David B. Kantor, Hitesh S. Sandhu, Saad Ghafoor

**Affiliations:** 1 Department of Pediatrics, Critical Care Medicine, The University of Tennessee Health and Science Center, Memphis, TN 38163, USA; eseewer@uthsc.edu (E.S.); wstaffo3@uthsc.edu (W.H.S.); nicolas.chiriboga.s@gmail.com (N.C.); tom.spentzas@gmail.com (T.S.); shyam.popat@bcm.edu (S.P.); alyssa.reece@lebonheur.org (A.C.); hsandhu@uthsc.edu (H.S.S.); saad.ghafoor@stjude.org (S.G.); 2 Department of Pediatric Medicine, St. Jude Children’s Research Hospital, Memphis, TN 38105, USA; 3 Department of Pediatrics, Critical Care Medicine, The University of South Alabama Women and Children’s Hospital, Mobile, AL 36604, USA; 4 Department of Anesthesiology, Critical Care, and Pain Medicine, Boston Children’s Hospital, Boston, MA 02115, USA; david.kantor@childrens.harvard.edu

**Keywords:** muscle loss, extracorporeal membrane oxygenation, pediatric critical care, point of care ultrasound, muscle wasting

## Abstract

Background: Critically ill children requiring extracorporeal membrane oxygenation (ECMO) support are at high risk of immobility, deconditioning, and muscle loss. There is a lack of screening and diagnostic tools to quantify muscle loss in this population. Objective: This study aims to evaluate the use of bedside ultrasound as a practical and effective method for detecting muscle loss in this high-risk group. Materials and Methods: This is a secondary analysis of a prospective observational clinical study conducted between January 2024 and January 2025 that used ultrasound to describe muscle loss in critically ill children aged 2 to 18 years. Results: The primary study enrolled 35 patients, five of whom required ECMO support. All patients who required ECMO showed significant muscle loss (>10%) in the quadriceps femoris, as measured by muscle thickness and cross-sectional area, compared with baseline measurements obtained before ECMO cannulation. Conclusions: Point-of-care muscle ultrasound could be a reliable, cost-effective tool for assessing muscle loss in pediatric patients on ECMO.

## 1. Introduction

Muscle loss in critically ill patients has deleterious consequences. It is a strong predictor of intensive care unit (ICU)-acquired weakness, which leads to prolonged hospital stay, prolonged duration of mechanical ventilation, and increased morbidity [1,2,3].

Bedside muscle ultrasound assessment has emerged as an attractive option for this purpose, as it is noninvasive and likely practical for screening for muscle loss in high-risk patients. Multiple studies involving both children and adults confirm that muscle loss can begin as early as 3–10 days following invasive mechanical ventilation and may strongly relate to clinical outcomes [4,5,6].

Extracorporeal membrane oxygenation (ECMO) is a life-saving technology increasingly used to treat patients with severe cardiac, respiratory, or combined cardiorespiratory failure [7]. Despite optimal ECMO support, patients often develop several ICU-related complications, including ICU-acquired muscle weakness and loss. These complications are usually related to multiple organ dysfunction, severity of illness, immobility, and prolonged hospital stay [8].

Children who require ECMO support often have life-threatening pathophysiology. The literature describes that, under these conditions, the body often functions in an extreme catabolic state, with negative protein balance and hyperglycemia. These conditions resemble the foundation for critical illness-induced polyneuropathy and muscle loss [9].

Initiating and maintaining ECMO is a complex process and requires a multidisciplinary approach and collaboration among multiple team members. Additionally, this population requires a high level of sedation and, at times, neuromuscular paralysis to ensure the safety of ECMO cannulas, especially in younger children, and to maintain adequate ECMO flows. This represents a significant challenge for providers, given the lack of ICU liberation and early physical activity bundles or protocols to guide such management [10]. This is speculated to correlate with deconditioning, muscle loss, and weakness, probably to a greater extent than in the general pediatric ICU population.

It has been suggested in the adult literature that patients requiring ECMO may present with worse muscle wasting and weakness compared with ICU patients of other etiologies, but there is no robust literature to support this hypothesis. In a prospective cohort study that assessed changes in quadriceps size in 25 adult ICU patients on ECMO using ultrasound measurements from baseline, the rectus femoris cross-sectional area decreased by 19% by day 10 and by 30.5% by day 20 [11]. Bear DE et al. also described that a low skeletal muscle index in patients with acute respiratory distress syndrome (ARDS) resulted in longer venovenous ECMO duration, while a preserved initial skeletal muscle index was associated with better survival [12].

Unfortunately, there is a lack of such studies in children and, additionally, a lack of accurate diagnostic and screening tools to describe muscle loss in this challenging population. We performed a single-center study using point-of-care ultrasound (POCUS) to assess muscle mass loss in 35 pediatric intensive care unit patients and performed a secondary analysis to describe muscle mass changes in a subset of patients who received ECMO support.

## 2. Materials and Methods

### 2.1. Study Design

This prospective cohort study was conducted in a 20-bed pediatric medical–surgical ICU at Le Bonheur Children’s Hospital, a freestanding tertiary care academic medical center affiliated with the University of Tennessee Health Science Center [13]. The University of Tennessee Health Science Center Institutional Review Board approved the study protocol (IRB #23-09784-XP) on 5 January 2024. Patients were enrolled from January 2024 to January 2025.

Pediatric patients meeting the following inclusion criteria were enrolled: aged 2–18 years with normal neurologic development and baseline gross motor function (including independent ambulation before hospitalization), no known neuromuscular disease, acute respiratory failure requiring endotracheal intubation, intubation duration of less than 72 h at enrollment, and anticipated need for mechanical ventilation exceeding 24 h. Written informed consent was obtained from parents or legal guardians, and patient assent was obtained when appropriate.

From electronic medical records, data on potential risk factors for muscle loss described in the literature, such as corticosteroid administration, neuromuscular blocking agent use, Pediatric Risk of Mortality III (PRISM-III) scores, and actual versus expected caloric and protein intake, were collected [14].

Subsequently, a subset of 5 patients who required either VA or VV ECMO support was analyzed separately to describe muscle loss in this patient cohort.

### 2.2. Muscle Measurements

All US measurements were performed by a licensed physical therapist and a pediatric critical care medicine clinical fellow, both of whom have specialized training in musculoskeletal US. Using a standardized protocol, the cross-sectional area (CSA) and muscle thickness (mT) of the quadriceps femoris muscle were assessed. Images were acquired using a SonoSite X-Porte US system (FUJIFILM SonoSite, Inc., Bothell, WA, USA) equipped with a C60xp curvilinear transducer (2–5 MHz). Measurements were obtained in B-mode, with patients positioned supine and limbs maintained in neutral alignment to ensure muscle relaxation.

Anatomical reference points were identified by palpation of the anterior superior iliac spine and the superior pole of the patella. The measurement site was standardized at two-fifths of the femoral length, measured from the anterior superior iliac spine. This location was marked with a sterile surgical skin marker (Medline Industries, Northfield, IL, USA) to ensure consistency across serial assessments. All participants underwent a baseline US assessment immediately upon enrollment in the study to establish reference values for mT and CSA. Protocol-mandated follow-up scans occurred at 72 h intervals until extubation.

### 2.3. Ultrasound Assessments

The US evaluation was initiated by positioning the C60xp curvilinear transducer (2–5 MHz) perpendicular to the skin surface in the short-axis orientation, at the pre-determined measurement site (two-fifths of the femoral length). The assessment site was selected to optimize visualization of the rectus femoris (RF), while accounting for potential obstructions that might compromise image quality.

**Figure 1:**
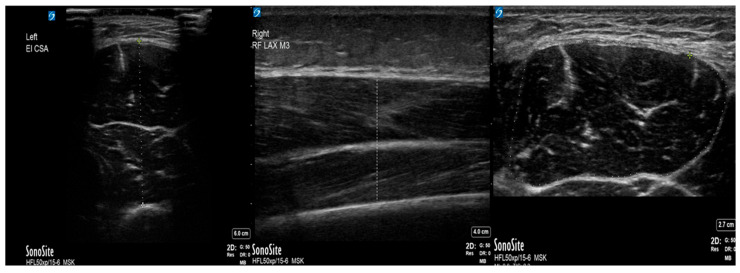
Representative ultrasound images of the quadriceps femoris muscle of a critically ill pediatric patient. Measures of the short axis (**left panel**), long axes (**middle panel**) and cross-sectional area (**right panel**) of the muscle were assessed.

Upon proper probe placement, the hyperechoic femoral cortex was identified, and images were acquired at the optimal point for tissue visualization. The mT was measured using the US system’s caliper tool, with measurement landmarks established between the superficial fascial border of the RF and the hyperechoic femoral cortex (Figure 1). The CSA was calculated using Sonosite’s area tool, tracing the outer fascia of the RF (Figure 1). All measurements were performed with careful attention to maintaining consistent probe pressure and orientation to ensure reproducibility. After the short-axis measurements were obtained, the transducer was rotated 90° to obtain long-axis images of the RF at the same anatomical landmark (Figure 1).

In this orientation, the mT was again measured between the superficial fascial border and the femoral cortex by using the digital caliper tool; CSA measurements were not acquired due to anatomical constraints inherent to the longitudinal view. To ensure measurement reliability, 2 independent mT measurements were obtained in both short- and long-axis orientations, and the mean values were calculated for the final analysis.

### 2.4. Outcomes

The primary measure was the percentage change in the quadriceps femoris muscle cross-sectional area and muscle thickness between the first and subsequent ultrasound assessments.

Significant muscle loss was defined as a drop in muscle thickness or cross-sectional area of >10% on any follow-up scan, according to the current literature.

### 2.5. Statistical Analysis

Continuous variables are presented as mean ± standard deviation (SD). Given the small sample size, 95% confidence intervals (CIs) for the mean were calculated using the student’s *t*-distribution. No formal hypothesis testing was performed due to the small sample size. A reduction of 10% or more in muscle thickness and cross-sectional area at any ultrasound assessment compared to the baseline was considered clinically significant. This cutoff aligns with prior studies that a reduction >10% is clinically meaningful [4,6].

## 3. Results

The primary study cohort consisted of 35 patients, of whom five required extracorporeal membrane oxygenation (ECMO) support and were included in this secondary analysis. Patient ages ranged from 5 to 16 years. Four patients received venovenous (VV) ECMO, while one patient received venoarterial (VA) ECMO (Table 1).

All patients underwent serial ultrasound assessments at four predefined time points: baseline (at enrollment) and every 72 h thereafter until extubation. Baseline measurements included quadriceps femoris muscle thickness and cross-sectional area.

**Figure 2:**
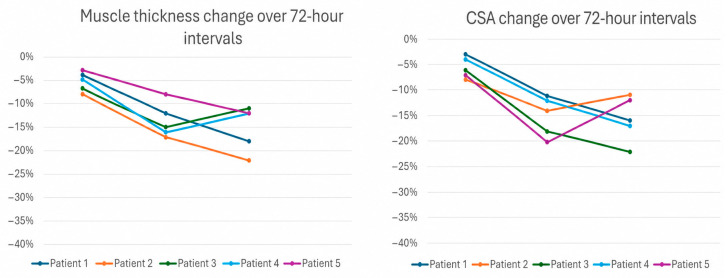
Trends of cross-sectional muscle area and muscle thickness over time.

Significant muscle loss was defined as a ≥10% reduction in muscle thickness on any follow-up scan. All patients demonstrated significant muscle loss during the study period. The degree of muscle loss ranged from 13% to 22%. The mean percentage muscle loss was −16.6% ± 3.6% (SD), indicating moderate interpatient variability. The estimated 95% confidence interval (CI) for mean muscle loss was −21.0% to −12.2%. Muscle loss was consistently observed using both quadriceps muscle thickness and cross-sectional area measurements (Figure 2).

Clinical risk factors associated with muscle loss—well described in the literature—were recorded for all patients, including length of stay, duration of mechanical ventilation, corticosteroid exposure, and PRISM III scores (Table 1). All patients required significant sedation with multiple agents. Additionally, 4 out of 5 patients required neuromuscular blockade for varying durations (Table 2). Nutritional intake was recorded for all patients, including patients’ actual caloric intake at the time of each ultrasound scan compared to their goal, and daily protein intake (Table 3).

## 4. Discussion

Currently, there is a lack of adequate screening or diagnostic tools to detect early muscle loss in children requiring ECMO support, and the fact that gold standards for measuring muscle mass, such as CT and MRI scans [15], are often not practical or portable—due to concerns of cannula dislodgment and catastrophic events—creates a challenge in assessing and quantifying potential muscle loss.

In this study, we describe a potentially practical, noninvasive approach utilizing point-of-care ultrasound to detect early muscle loss in this population. Quantitative assessment of quadriceps femoris muscle thickness and cross-sectional area enabled the identification of muscle loss within our limited patient cohort across varying stages of illness, occurring as early as 3–10 days following the initiation of mechanical ventilation and ECMO support. These findings are consistent with prior studies employing similar ultrasound-based methodologies in the general PICU population [4,5,6].

This supports the current body of literature in adults, which shows that ECMO support, though a very effective intervention to sustain life in severe illness, could be associated with increased ICU-acquired weakness and muscle loss [16]. This could be related to delays in early physical therapy, heavy sedation, and prolonged neuromuscular blockade.

The current literature suggests that early implementation of physical therapy shortens the length of stay and improves clinical outcomes in critically ill children [17]; however, such data remain lacking for the pediatric ECMO population. Reasons behind this gap in research includes safety concerns and cannula dislodgment events. A few alternatives have been proposed in the literature when it is not feasible or practical to implement physical therapy early in the course of critical illness, such as electrical muscle stimulation, which shows some promising data regarding improved functional strength scores and outcomes [18].

Data on optimizing nutrition for critically ill children, including early initiation of total parenteral nutrition and increased protein intake, remain controversial. Stacey et al. recently published a meta-analysis evaluating the relationship between changes in muscle mass and protein or energy intake in critically ill children and reported that data across different nutritional strategies were inconsistent [19].

Finally, follow-up of children who received ECMO support shows that a significant percentage of survivors suffered from motor disabilities, with delayed or no return to their normal baseline prior to illness, and a prolonged need for outpatient occupational and physical therapy. However, studies describing muscle mass on follow-up are currently lacking [20,21].

## 5. Limitations

This study represents a secondary analysis of a small sample of patients conducted at a single center, which clearly hampers generalizability. Additionally, its observational nature does not allow statistical analysis to establish causality or compare rates of loss with non-ECMO patients. The ultrasound technique, though standardized, is operator dependent.

This suggests the need for larger-scale studies to establish patterns of early muscle loss in ECMO patients across multiple centers, identify risk factors, and potentially implement early physical therapy and optimal nutrition strategies to protect against early muscle loss and deconditioning in such a vulnerable population.

## 6. Conclusions

Despite its limitations, this study makes a compelling case. Point-of-care ultrasound could be a feasible and practical tool for detecting early, significant muscle loss in pediatric patients on ECMO. This technique holds immediate promise as a monitoring tool, giving clinicians eyes into a previously hidden aspect of their patients’ physiology. We believe it could serve as a vital diagnostic tool to guide early, targeted interventions to mitigate the burden of ICU-acquired weakness.

Ultimately, validating these findings in larger, prospective studies that link ultrasound metrics to functional outcomes is an essential next step toward improving the long-term recovery of these most vulnerable children.

## Figures and Tables

**Table 1:** Clinical characteristics of patients enrolled in the study. Abbreviations: ECMO, extracorporeal membrane oxygenation; VV, venovenous; VA, venoarterial; PICU, pediatric intensive care unit; ARDS, acute respiratory distress syndrome; PRISM, Pediatric Risk of Mortality.

Patient	Age (Years)	Sex	Indication for ECMO	ECMO Mode	PICU Stay (Days)	Intubation Duration (Days)	PRISM III Score
P1	14	M	Respiratory arrest due to status asthmaticus and bilateral tension pneumothorax	VV	14	10	4
P2	16	F	Severe ARDS secondary to multifocal pneumonia and vasculitis	VV	21	14	8
P3	16	M	Gunshot wound to the chest with pulmonary contusions	VV	35	16	8
P4	5	F	Severe ARDS following bone marrow transplantation for neuroblastoma	VV	18	15	28
P5	7	M	Refractory septic shock	VA	20	12	22

**Table 2:** Description of sedation and neuromuscular blockade use in enrolled patients.

Patient	Corticosteroids (Total Dose, Duration)	Midazolam (Total Dose, Duration)	Fentanyl (Total Dose, Duration)	Dexmedetomidine (Total Dose, Duration)	Neuromuscular Blockade (Days)
P1	Methylprednisolone, 1141 mg over 14 days	548 mg over 6 days	22,650 µg over 10 days	17,482.8 µg over 13 days	4
P2	Methylprednisolone, 4590 mg over 19 days	Not administered	50,830 µg over 15 days	39,249 µg over 17 days	3
P3	Dexamethasone, 4 mg (single dose)	556.5 mg over 10 days	33,950 µg over 13 days	20,807.1 µg over 15 days	0
P4	Methylprednisolone, 60 mg + hydrocortisone, 335.5 mg over 12 days	503.1 mg over 8 days	1866 µg over 8 days	2847.8 µg over 8 days	8
P5	Hydrocortisone, 200 mg (duration not specified)	350 mg over 7 days	1355 µg over 12 days	1442 µg over 9 days	2

**Table 3:** Recorded muscle loss based on the cross-sectional area (CSA), and muscle thickness of the quadriceps femoris muscle in relation to nutritional status. Abbreviations: MT, muscle thickness; CSA, cross-sectional area.

Patient	Scan	MT (cm)	ΔMT (%)	CSA (cm^2^)	ΔCSA (%)	Caloric Intake (% of Requirement)	Protein Intake (g/kg/day)
P1	1	3.90	–	9.30	–	–	–
	2	3.75	−4	9.02	−3	50	1.38
	3	3.43	−12	8.28	−11	75	1.46
	4	3.19	−18	7.81	−16	66	1.42
P2	1	2.30	–	7.20	–	–	–
	2	2.12	−8	6.62	−8	33	1.40
	3	1.91	−17	6.19	−14	45	1.38
	4	1.79	−22	6.40	−11	88	1.41
P3	1	4.80	–	15.30	–	–	–
	2	4.47	−7	14.38	−6	11	0.27
	3	4.08	−15	12.54	−18	23	1.35
	4	4.27	−11	11.93	−22	78	1.83
P4	1	1.60	–	1.80	–	–	–
	2	1.52	−5	1.73	−4	24	2.48
	3	1.34	−16	1.58	−12	58	1.97
	4	1.40	−12	1.49	−17	75	2.23
P5	1	1.80	–	2.10	–	–	–
	2	1.74	−3	1.95	−7	28	1.20
	3	1.65	−8	1.68	−20	72	1.40
	4	1.58	−12	1.76	−16	92	1.70

## Data Availability

The original contributions presented in this study are included in the article. Further inquiries can be directed to the corresponding authors.
